# RETRACTED: Cui et al. Impact of High-Frequency Traveling-Wave Magnetic Fields on Low-Conductivity Liquids: Investigation and Potential Applications in the Chemical Industry. *Materials* 2024, *17*, 944

**DOI:** 10.3390/ma17215270

**Published:** 2024-10-30

**Authors:** Xinyu Cui, Xianzhao Na, Xiaodong Wang, Roland Ernst, Fautrelle Yves

**Affiliations:** 1 State Key Laboratory of Advanced Steel Processing and Products, Central Iron and Steel Research Institute, Beijing 100081, China; cuixinyu0626@163.com; 2 College of Material Science and Opto-Electronic Technology, University of the Chinese Academy of Sciences, Beijing 100049, China; 3 Université Grenoble Alpes, CNRS, Grenoble INP, SIMAP, F-38000 Grenoble, France

The journal retracts the article titled “Impact of High-Frequency Traveling-Wave Magnetic Fields on Low-Conductivity Liquids: Investigation and Potential Applications in the Chemical Industry” [1], cited above.

Following publication, the authors contacted the Editorial Office to raise concerns relating to the accuracy of the original authorship list and the presence of scientific errors within this study [1]. 

Adhering to our standard procedure, the Editorial Office and Editorial Board conducted an investigation that confirmed that a number of originally listed authors were not aware of this submission, nor were they provided with the opportunity to critically review the findings presented. Furthermore, following further discussions with the authors, the Editorial Board confirmed the presence of significant scientific interpretation and methodological flaws within the presented findings. As a result, the Editorial Office, Editorial Board, and authors have decided to retract this article as per MDPI’s retraction policy (https://www.mdpi.com/ethics#_bookmark30, accessed on 13 May 2024). 

This retraction was approved by the Editor-in-Chief of the *Materials* journal.

The authors agreed to this retraction.
